# Vaginal Mucositis in Measles

**DOI:** 10.1155/S1064744995000184

**Published:** 1995

**Authors:** Ingrid A. Chamales, Peter G. Napolitano, Cesar Rosa

**Affiliations:** 1 Department of Obstetrics and Gynecology William Beaumont Army Medical Center El Passo TX USA; 2 Division of Maternal-Fetal Medicine University of Florida College of Medicine P.O. Box 100294 Gainesville FL 32610-0294 USA

## Abstract

*Background:* Measles (rubeola), a common childhood exanthema, occurs infrequently in adults. Vaginal mucositis in association with measles is not commonly described.

*Case:* During a recent measles epidemic, 2 female patients presented with high fever, myalgia, exanthema, and prostration. On examination, each patient had marked inflammation and tenderness of the vaginal mucosa, prompting the presumptive diagnosis of toxic shock syndrome. The evolution of the illness was consistent with measles. Cervicovaginal cultures were negative for pathogens. Acute and convalescent antibody titers for Rocky Mountain spotted fever, rubella, leptospirosis, and *Proteus* Ox-19 were not consistent with a recent infection. The sera also were negative for anti-toxic shock toxin-1 and anti-streptolysin. Measles antibody titers were consistent with a recent infection.

*Conclusion:* Vaginal mucositis is an unusual manifestation of measles that may mimic toxic shock syndrome.


Infectious Diseases in Obstetrics and Gynecology 2:279–281 (1995)
© 1995 Wiley-Liss, Inc.

### Vaginal Mucositis in Measles

Ingrid A. Chamales, Peter G. Napolitano, and Cesar Rosa

*Department of Obstetrics and Gynecology, William Beaumont Army Medical Center, El Passo, TX*

ABSTRACT

***Background:*** **Measles (rubeola), a common childhood exanthema, occurs infrequently in adults. Vaginal mucositis in association with measles is not commonly described.**

***Case:*** **During a recent measles epidemic, 2 female patients presented with high fever, myalgia, exanthema, and prostration. On examination, each patient had marked inflammation and tenderness of the vaginal mucosa, prompting the presumptive diagnosis of toxic shock syndrome. The evolution of the illness was consistent with measles. Cervicovaginal cultures were negative for pathogens. Acute and convalescent antibody titers for Rocky Mountain spotted fever, rubella, leptospirosis, and *Proteus* Ox-19 were not consistent with a recent infection. The sera also were negative for anti-toxic shock toxin-1 and anti-streptolysin. Measles antibody titers were consistent with a recent infection.**

***Conclusion:*** **Vaginal mucositis is an unusual manifestation of measles that may mimic toxic shock syndrome.** © 1995 Wiley-Liss, Inc.*

KEY WORDS

Koplik spots, rubeola, toxic shock syndrome, vaginal inflammation, vaginal erythema

Measles, a common childhood viral exanthema, is now uncommon due to a successful vaccination program. The usual clinical manifestations are malaise, fever, conjunctivitis, hacking cough, rhinitis, and a characteristic centrifugal maculopapular, erythematous rash. Koplik spots, a characteristic mucous-membrane finding presenting as small, irregular, slightly raised white lesions, precede the onset of the rash by 1–2 days. They have the appearance of grains or clumps of salt on a red background (Fig. 1).[1] On histology, Koplik spots consist of cytoplasmic and intranuclear inclusions, giant cells, and intercellular edema.[2] Though most commonly found on the buccal mucosa opposite the molars, Koplik spots have been described on the pharynx and tracheobronchial mucosa.[2] We could find only one publication in the medical literature that referred to vaginal Koplik spots.[3] Vaginal mucositis associated with measles has not been reported previously, possibly because, heretofore, most patients affected with measles were prepubertal. We review the cases of 2 female patients of reproductive age who presented with vaginal mucositis and measles. We believe that this may be the first report of this clinical finding in association with measles.

### CASE REPORTS

Patient 1, a 21-year-old $G_1$ $P_{1001}$, presented with complaints of a sore throat with a nonproductive cough, fever, chills, and myalgia of 3 days duration. She was menstruating and using vaginal tampons. No other family members were ill, and she was not aware of any recent exposure to insect bites or infectious diseases. She had received all of the childhood vaccinations. On examination, her vital

**Address correspondence/reprint requests to Dr. Cesar Rosa, Division of Maternal-Fetal Medicine, University of Florida College of Medicine, P.O. Box 100294, Gainesville, FL 32610-0294.**

**The opinions or assertions contained herein are the private views of the authors and are not to be construed as official or as reflecting the views of the Department of the Army or the Department of Defense.**

***This article is a U.S. Government work and, as such, is in the public domain in the United States of America.**

Received October 12, 1994
Accepted January 12, 1995

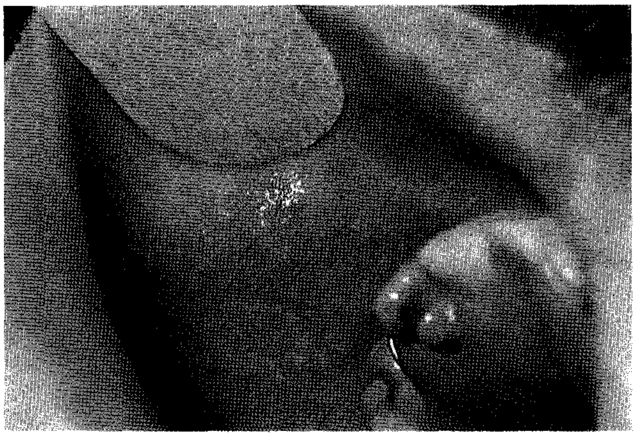

Fig. 1. Koplik spots on the buccal mucosa. (Reproduced from Forbes CD, Jackson WF: A Colour Atlas and Text of Clinical Medicine. London: Mosby-Yearbook Europe Ltd., 1993. With permission.)

signs were stable. Her temperature was 39.4°C. The relevant findings included a facial maculopapular rash, conjunctival injection, coryza, and cervical adenopathy. The pharynx was erythematous, and Koplik spots were seen on the buccal mucosa. On pelvic examination, she had pain upon insertion of the speculum. The vaginal vault contained blood consistent with her menstruation, and the vaginal mucosa appeared intensely erythematous. No purulent discharge was observed, and bimanual examination revealed no masses. Her diffuse pelvic pain was attributed to the mucosal inflammation. The WBC count was 4,500/mm$^3$, and the erythrocyte sedimentation rate was 8 mm/h. The urinalysis was normal, and a chest X-ray was normal. Blood, cervicovaginal, and throat cultures were negative. She was admitted to the hospital with a presumptive diagnosis of toxic shock syndrome and was treated with IV antibiotics. Over the next 3 days, the rash extended over her trunk and extremities. The clinical presentation and evolution, the antibody assays described below, and a measles outbreak in the community rendered measles as the diagnosis.

Patient 2, a 31-year-old $G_6$ $P_{4024}$, presented 24 h later with similar symptoms. A rash had started to develop 4 days previously. She also was menstruating, but was not using vaginal tampons. She had received a measles, mumps, and rubella (MMR) vaccination in 1963. On examination, her vital signs were stable and her temperature was 39.8°C. A maculopapular rash was present on the face, neck, and trunk. Koplik spots were present on the buccal mucosa. There was cervical adenopathy, and the lung fields were clear on auscultation. The pelvic examination revealed marked vulvovaginal hyperemia, with Koplik spots on the vaginal mucosa. There was no tenderness on a bimanual examination. The WBC count was 3,900/mm$^3$, and a chest X-ray was normal. Blood, cervicovaginal, and throat cultures were negative. She was admitted to the hospital for IV hydration and close observation.

The possibility of toxic shock syndrome was strongly considered in both patients, given the fever, rash, vaginal inflammation, and temporal relation with menstruation, but was ruled out on the basis of negative assays for anti-toxic shock toxin-1. Both patients had negative genital cultures for gonorrhea, chlamydia, group A and B streptococcus, and staphylococcus.

In an effort to better define the nature of the illness, we obtained acute and convalescent titers for the following conditions: toxic shock syndrome, Rocky Mountain spotted fever, group A streptococcal infection, typhus, leptospirosis, rubella, and measles. Serum specimens were negative for anti-toxic shock toxin-1, Rocky Mountain spotted fever, and leptospirosis. Acute and convalescent sera were unchanged for anti-streptolysin, *Proteus* Ox-19, and rubella. Patient 1 had a 4-fold increase in measles IgG antibodies as measured by hemagglutination inhibition. Patient 2 did not show a change in titer, with the paired sera both showing a titer of 1:128. At the time, we did not have access to a measles IgM antibody assay. In spite of the unchanged titers, patient 2's clinical presentation and the evolution of her disease were so suggestive of measles that, in the opinions of the dermatology and infectious disease consultants, measles did occur. It is our hypothesis that patient 2 might have had an anamnestic IgG response following her previous (1963) vaccination with a killed virus and that she had reached peak antibody levels at the time of the initial blood draw.

### CONCLUSIONS

Measles is a common childhood disease, but it occurs infrequently in adults. At the time that these 2 patients presented for care, there was an outbreak of measles in the community. The county department of health had received reports of 55 adult (over 20 years of age) cases between December

1989 and May 1990. Thirty-one of those affected had been vaccinated previously against measles (El Paso County Health Education and Epidemiology, personal communication).

Of interest, most people born before 1957 have been exposed to measles and are now immune. Starting in 1963, 2 measles vaccines were dispersed: one made from a live attenuated virus and the other from a killed virus. The latter was distributed until 1967, when its association with atypical measles was established. When exposed to measles, those who were vaccinated with the killed virus vaccine were prone to develop severe pneumonia, high fever, and extreme prostration.[4] We believe that patient 2, and maybe patient 1, had an episode of atypical measles.[5] The recommendation stands that individuals be revaccinated if they were born after 1957 and cannot document previous live virus vaccination or serologic evidence of immunity.[2]

An intense vaginal mucositis in association with measles is a heretofore unreported finding. With an estimated 5% of the population unvaccinated and at risk of contracting measles, sporadic outbreaks of measles are likely to continue. As has been the recent trend, the affected population will likely be skewed to the older age group,[6] thus placing the gynecologist in a position to identify those patients who may present with vaginal mucositis.

Koplik spots, the pathognomonic lesions of measles, fade rapidly and are difficult to see after 3 days. To our knowledge, this report is only the second in the medical literature to describe vaginal Koplik spots. We propose that the presence of vaginal Koplik spots may be helpful in diagnosing vaginal mucositis of measles. Because of its potentially fatal complications, toxic shock syndrome should always be considered in any female presenting with vaginal mucositis and excluded by appropriate tests.

### REFERENCES

1. Bernstein DJ, Schiff GM: Measles. In Gorbach SL, Bartlett JG, Blacklow NR (eds): Infectious Diseases. Philadelphia: W.B. Saunders, pp 1088–1092, 1992.
2. Ray CG: Measles. In Isselbacher KJ, Braunwald E, Wilson JD, Martin JB, Fauci AS, Kasper DL (eds): Harrison's Principles of Internal Medicine. New York: McGraw-Hill, pp 825–827, 1994.
3. Baraff L: Measles. In Thadepalli H (ed): Infectious Diseases: Focus on Clinical Diagnosis. New York: Medical Examination Press, pp 664–666, 1980.
4. Fulginiti VA, Eller JJ, Downie AW, Kempe CH: Altered reactivity to measles virus: Atypical measles in children previously immunized with inactivated measles virus vaccines. JAMA 202:1075–1080, 1967.
5. Cherry JD, Feigin RD, Lobes LA, Shakelford PG: Atypical measles in children previously immunized with attenuated measles vaccines. Pediatrics 50:712–717, 1972.
6. Centers for Disease Control: Current trends: Measles—United States, first 26 weeks, 1994. MMWR 43(37): 673–676, 1994.
